# Deep Learning for Accurate Segmentation of Venous Thrombus from Black-Blood Magnetic Resonance Images: A Multicenter Study

**DOI:** 10.1155/2021/4989297

**Published:** 2021-12-14

**Authors:** Chuanqi Sun, Xiangyu Xiong, Tianjing Zhang, Xiuhong Guan, Huan Mao, Jing Yang, Xiaoyong Zhang, Yi Sun, Hao Chen, Guoxi Xie

**Affiliations:** ^1^Department of Biomedical Engineering, The Sixth Affiliated Hospital, Guangzhou Medical University, Guangzhou, China; ^2^Philips Healthcare, Guangzhou, China; ^3^Siemens Healthcare, Shanghai, China; ^4^Jiangsu JITRI Sioux Technologies Co., Ltd., Suzhou, China

## Abstract

**Objective:**

Deep vein thrombosis (DVT) is the third-largest cardiovascular disease, and accurate segmentation of venous thrombus from the black-blood magnetic resonance (MR) images can provide additional information for personalized DVT treatment planning. Therefore, a deep learning network is proposed to automatically segment venous thrombus with high accuracy and reliability.

**Methods:**

In order to train, test, and external test the developed network, total images of 110 subjects are obtained from three different centers with two different black-blood MR techniques (i.e., DANTE-SPACE and DANTE-FLASH). Two experienced radiologists manually contoured each venous thrombus, followed by reediting, to create the ground truth. 5-fold cross-validation strategy is applied for training and testing. The segmentation performance is measured on pixel and vessel segment levels. For the pixel level, the dice similarity coefficient (DSC), average Hausdorff distance (AHD), and absolute volume difference (AVD) of segmented thrombus are calculated. For the vessel segment level, the sensitivity (SE), specificity (SP), accuracy (ACC), and positive and negative predictive values (PPV and NPV) are used.

**Results:**

The proposed network generates segmentation results in good agreement with the ground truth. Based on the pixel level, the proposed network achieves excellent results on testing and the other two external testing sets, DSC are 0.76, 0.76, and 0.73, AHD (mm) are 4.11, 6.45, and 6.49, and AVD are 0.16, 0.18, and 0.22. On the vessel segment level, SE are 0.95, 0.93, and 0.81, SP are 0.97, 0.92, and 0.97, ACC are 0.96, 0.94, and 0.95, PPV are 0.97, 0.82, and 0.96, and NPV are 0.97, 0.96, and 0.94.

**Conclusions:**

The proposed deep learning network is effective and stable for fully automatic segmentation of venous thrombus on black blood MR images.

## 1. Introduction

Deep vein thrombosis (DVT) is now the third-largest cardiovascular disease after cerebral vascular and coronary artery disease, it occurs mainly in the lower extremities, and with the acceleration of population aging, the incidence rate of DVT is increasing year by year [1]. Failure to accurately diagnose DVT can lead to severe complications, such as postthrombotic syndrome, pulmonary embolism, lower extremity venous ulcer, and chronic pulmonary hypertension [2].

Recently, an MR black-blood thrombus imaging (BTI) technique was developed to diagnose DVT [3]. The technique uses a black-blood preparation to suppress the venous blood flow signals and thus make the thrombus be directly visualized within the black-blood venous lumen. Some studies have demonstrated that BTI is reliable and accurate for diagnosing DVT without the use of contrast agents [4, 5]. Accurate quantification of thrombus characteristics, such as thrombus distribution, signal intensities, volume, and shape, can provide additional information for personalized DVT treatment planning [6, 7]. However, the key step to quantify these thrombus characteristics is to segment the thrombus from BTI images, which remains a challenging task at present.

**Figure 1:**
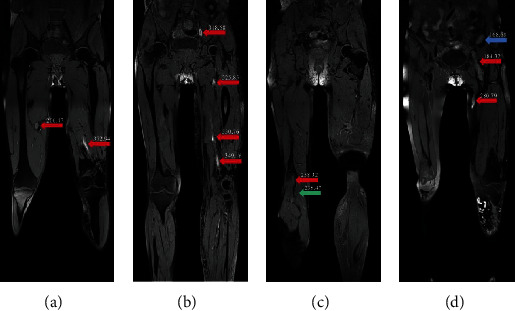
Representative images are obtained by BTI from four DVT patients. Images are shown with the same window and level, and the value on the arrow represents the signal intensity. (a) Different signal intensities of the thrombus (red arrows) can be observed in a patient; (b) the thrombus (red arrows) can distribute in a large area from the common iliac vein to the popliteal vein; (c) the thrombus (red arrow) may have very similar signal intensity with the adjacent muscle (green arrow); (d) iliac tissues (blue arrow) may have the similar signal intensity and shape with the thrombus (red arrow).

The challenges of segmenting the thrombus from BTI images are due to the following reasons. Firstly, a complete BTI scan for a DVT patient can obtain hundreds of images, which makes manually contouring the thrombus from such a lot of images a tremendously difficult task. Secondly, the thrombus characteristics on BTI images hinder the performance of automatic segmentation approaches that can be directly employed on this task. These characteristics include (1) thrombus signal intensities vary in different patients and even in the same patient (Figure 1(a)), leading to difficulty in segmenting thrombus accurately; (2) thrombus locations are in a large area and random in a patient (Figure 1(b)), the unfixed thrombus locations make the segmentation work more difficult; (3) other tissues may have the very similar signal intensity and shape with the thrombus (Figures 1(c) and 1(d)), leading to the high possibility that other tissues can be misidentified as the thrombus.

In recent years, deep learning network has demonstrated enormous potential in the field of medical image segmentation [8–10]. Some automatic segmentation methods have been applied to aortic thrombus segmentation on postoperative computerized tomography angiography images [11, 12]. However, to the best of our knowledge, there are not any reports about automatic segmentation of venous thrombus on black blood MR images. Therefore, we develop a fully automatic method of venous thrombus segmentation based on deep learning network and BTI images, aiming to reduce the burden of clinicians and improve the efficiency and accuracy of DVT personalized treatment planning. The main contributions of this work are (1) three-dimensional (3D) U-shape segmentation model is incorporated into generative adversarial network (GAN) architecture for achieving accurate thrombus segmentation, especially to distinguish the tissues which may be misidentified as thrombus; (2) this work is the first attempt for the thrombus segmentation from BTI images, which can assist in making a personalized and accurate treatment plan for DVT patients; (3) the developed network has been extensively compared with state-of-the-art alternatives on the testing and other two external testing sets, which demonstrates that our proposed network achieves high accuracy and stability on the thrombus segmentation comprehensively.

## 2. Materials and Methods

### 2.1. Data Collection

Total images of 110 subjects are obtained from three different centers using two different BTI techniques. One of the BTI techniques is DANTE-SPACE which uses delay alternating with nutation for tailored excitation (DANTE) for black-blood preparation and a 3D variable flip angle fast spin echo sequence (SPACE) for readout [13]. The other is DNATE-FLASH which also uses DANTE for black-blood preparation but a fast low-angle shot sequence (FLASH) for readout [14]. 85 subjects used for network training and testing are obtained from one center using DANTE-SPACE on 1.5 T MR scanners (SIEMENS Avanto, Healthcare, Erlang, Germany). 15 and 10 subjects used for external testing are obtained from other two centers using DANTE-SPACE on a 3.0 T (SIEMENS Trio, Erlang, Germany) and DANTE-FLASH on a 3.0 T (SIEMENS Skyra, Erlang, Germany) MR scanners, respectively. In order to cover the whole lower extremities, two- or three-station scans are performed using a 6-channel body coil, an 8-channel external vascular coil, and a corresponding integrated spine coil. Scan parameters are shown in Table 1. After the scans are completed, the images are then composited into a large volumetric dataset using postprocessing software (SIEMENS Syngo, Germany). The volumetric dataset covering the whole lower extremities is finally used for thrombus segmentation.

### 2.2. Data Preprocessing

The ground truth of thrombus lesions is manually contoured with the consensus between two experienced radiologists on the BTI images. To enable our proposed network to learn venous thrombus features properly, all patients are resampled to the median voxel spacing of their respective dataset, where third-order spline interpolation is used for image data and nearest-neighbor interpolation for the corresponding ground truth. Then, the *z*-score normalization is applied to guarantee that the grey values of the same tissue among different MR images are close to each other [15]. Input images are cropped with a sliding window approach, in which the window size equals the patch size (height × width × channel, 192 × 160 × 64) used during network training, and adjacent images overlap by half of the size of a patch. Random rotations, random scaling, and elastic deformations are applied to increase the dataset size to avoid overfitting.

### 2.3. Deep Learning Network Architecture

**Figure 2:**
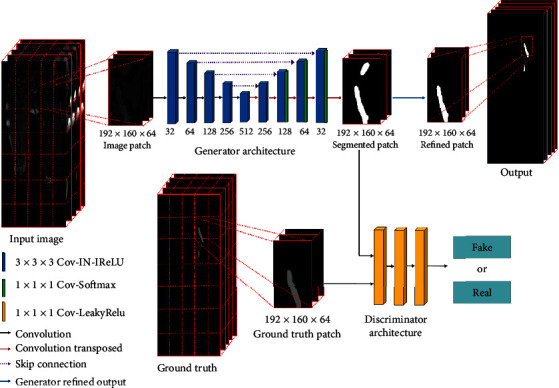
The workflow of the proposed network for the thrombus segmentation from BTI images. 3D segmentation model is used as the generator and its segmented patch and ground truth patch are fed into the discriminator to refine the segmentation result.

The deep learning network based on generative adversarial network (GAN) is proposed for automatic venous thrombus segmentation (as shown in Figure 2). The three-dimensional (3D) U-shape segmentation model is trained as the generator, and the 3D discriminator jointly supervises the segmentation performance in the proposed network. This strategy forces the network to train a more powerful generator to avoid segmentation errors. The proposed segmentation network is implemented by a 3D end-to-end patch-based GAN model (image patch size, 192 × 160 × 64), which takes the lower extremity black blood MR images as input and outputs equal-sized venous thrombus segmentation. Firstly, in the generator stage, the architecture of 3D U-shape generator contains encoding and decoding phases. The encoding phase with downconvolution blocks is used to extract the feature information of venous thrombus, and the decoding phase with upconvolution blocks is adopted to reconstruct the feature maps to probability maps. All the down- and upconvolution blocks contain 3 × 3 × 3 convolution, instance normalization, and Leaky ReLu layer. Furthermore, the last three upconvolution blocks contain 1 × 1 × 1 convolution and softmax layer additionally. Between each downconvolution block and corresponding upconvolution block, skip connection layers are applied for feature fusion of high- and low-resolution feature maps. Also, the combination of cross-entropy loss and soft dice loss is adopted as the loss function in the generator. Secondly, in the discriminator stage, the 3D discriminator is used to judge the authenticity of generated segmentation results against the reference manual ground truth, which contains three 1 × 1 × 1 convolution layers and uses Leaky ReLu as the activation function. The generator and discriminator networks are optimized one after the other in a zero-sum game framework. The generator's training objective is to increase the judgment error of the discriminator by producing novel segmented venous thrombus that is indistinguishable from the ground truth. The discriminator's training objective is to decrease the judgment error of the discriminator network and enhance the ability of differentiating the real from the fake. Adversarial loss is applied in both networks so that the generator produces more realistic segmentation, while the discriminator becomes more skilled at flagging segmented venous thrombus against ground truth. Finally, the refined patch of the generator will be restored to the original size according to the patch location and voxel spacing.

### 2.4. Loss Function

The total objective function (*L*_total_) in the developed network is the summation of the losses from generator and discriminator as

(1) $$\begin{array}{c} L_{\text{total}}=\lambda_{1}L_{\text{CE}}+\lambda_{2}L_{\text{SD}}+\lambda_{3}L_{\text{GAN}}, \end{array}$$

where *λ*_1_, *λ*_2_, and *λ*_3_ are weights, *L*_CE_, *L*_SD_, and *L*_GAN_ are cross-entropy loss, soft dice loss, and GAN loss, respectively. They are defined as

(2) $$\begin{array}{c} L_{\text{CE}}=\overset{N}{\underset{i=1}{\sum }}\left(y_{i}\cdot \log x_{i}+\left(1-y_{i}\right)\cdot \log\left(1-x_{i}\right)\right), \end{array}$$

(3) $$\begin{array}{c} L_{\text{SD}}=1‐\frac{2\sum_{i=1}^{N}x_{i}y_{i}}{\sum_{i=1}^{N}{x_{i}}^{2}+\sum_{i=1}^{N}{y_{i}}^{2}}, \end{array}$$

(4) $$\begin{array}{c} L_{\text{GAN}}=L_{\text{MSE}}\left\{{Ε}_{y}\left[\log D_{\theta_{D}}\left(y\right)\right]+{Ε}_{x_{i}}\left[\log\left(1-D_{\theta_{D}}\left(G_{\theta_{G}}\left(x_{i}\right)\right)\right)\right]\right\}, \end{array}$$

where *x*_*i*_ denotes the predicted probability of the *i*th pixel, *y*_*i*_ is the corresponding ground truth, and *N* is the number of pixels. *L*_MSE_ is the mean square error (MSE); *θ*_*G*_ and *θ*_*D*_, respectively, represent the parameters for the generator and discriminator in GAN. The *L*_CE_ is used to describe the distance of probability distributions between segmented venous thrombus and ground truth. Employing *L*_CE_ can segment high intensity and large area thrombus clearly [16]. *L*_SD_ is used here to solve the data imbalanced exists in the whole lower extremity MR images because the size of some venous thrombus is much smaller than background [17]. Due to the similar intensity and shape between thrombus and other tissue, *L*_GAN_ is used to identify thrombus areas strictly, and it learns the mapping from the input black blood MR images to ground truth [18].

### 2.5. Network Training and Testing

This work is performed using Pytorch on a workstation equipped with an NVIDIA TITAN V 12G GPU. During the network training and testing, 85 subjects obtained by DANTE-SPACE from the 1.5 T MR scanner are randomly divided into five groups for 5-fold cross-validation strategy. For each cross-validation, four groups of patients are used as training sets, and the remaining group is a testing set. 15 and 10 subjects obtained from the other two centers are used for external testing. During training, the parameters are set as follows: basic learning rate, 3 × 10^−4^; batch size, 2; and Adam is performed as optimization. After adequate training on the network, testing sets are used to test the network's performance.

### 2.6. Evaluation Metrics

Metrics based on pixel and vessel segment level are adopted to evaluate the difference between segmentation results and ground truth. The pixel level evaluation metrics, including dice similarity coefficient (DSC), absolute volume difference (AVD), and average Hausdorff distance (AHD), are given by

(5) $$\begin{array}{c} \text{DSC}=\frac{2\text{TP}}{2\text{TP}+\text{FP}+\text{FN}}, \end{array}$$

(6) $$\begin{array}{c} \text{AVD}=\frac{\;|\;\text{GT}‐P\;|\;}{\text{GT}}, \end{array}$$

(7) $$\begin{array}{c} \text{AHD}=\frac{1}{\;|\;\text{GT}\;|\;}\left(\underset{gt\in \text{GT}}{\sum }d\left(gt,P\right)\right), \end{array}$$

where TP denotes the true positive, FP denotes false positive, FN denotes false negative, P denotes prediction, GT denotes ground truth, *p* and *gt* are two points in P and GT, and *d* denotes Hausdorff distance.

The deep veins of one leg can be divided into 13 vessel segments, including inferior vena cava, common iliac vein, internal iliac vein, external iliac vein, common femoral vein, deep femoral vein, superficial femoral vein, popliteal vein, anterior tibial vein, posterior tibial vein, fibular vein, great saphenous vein, and small saphenous vein [19]. As correctly identifying which vessel segment occurs thrombosis is important for treatment-decision making [20]. The evaluation metrics based on vessel segment level include sensitivity (SE), specificity (SP), accuracy (ACC), positive predictive value (PPV), and negative predictive value (NPV). These metrics can be calculated according to Equations (8)–(12), respectively.

(8) $$\begin{array}{c} \text{SE}=\frac{\text{TP}}{\text{TP}+\text{FN}}, \end{array}$$

(9) $$\begin{array}{c} \text{SP}=\frac{\text{TN}}{\text{TN}+\text{FP}}, \end{array}$$

(10) $$\begin{array}{c} \text{ACC}=\frac{\text{TP}+\text{TN}}{\text{TP}+\text{FP}+\text{TN}+\text{FN}}, \end{array}$$

(11) $$\begin{array}{c} \text{PPV}=\frac{\text{TP}}{\text{TP}+\text{FP}}, \end{array}$$

(12) $$\begin{array}{c} \text{NPV}=\frac{\text{TN}}{\text{TN}+\text{FN}}, \end{array}$$

where TP, FP, TN, and FN are true positive, false positive, true negative, and false negative based on the vessel segment level.

### 2.7. Ablation Experiment and Parameter Sensitivity Analysis

In this work, we select and combine *L*_CE_, *L*_SD_, and *L*_GAN_ in our proposed network, and then, we did an ablation experiment to employ different loss function in the network (seen as below Table 2). Results in the table demonstrate that the loss functions combination is capable of improving the performance of segmentation.

After the loss function ablation experiment, we did a more detailed parameter sensitivity analysis. We defined *L*_total_ = *λ*_1_*L*_CE_ + *λ*_2_*L*_SD_ + *λ*_3_*L*_GAN_, *λ*_1_, *λ*_2_, and *λ*_3_ are weights; these are empirically set to 0.4, 0.6, and 1.0 based on the observation on the validation set. We have done multiple experiments on the adjustment of these weights and show some representative parameter results here (see Table 3). As the discriminative strategy is essential in the proposed network, we first select the correct *λ*_3_ value for the GAN loss; secondly, we keep *λ*_3_ and *λ*_1_ values unchanged, and adjust the appropriate *λ*_2_ value; finally, we choose the most suitable *λ*_1_ value to get the best segmentation performance.

## 3. Results

The proposed deep learning network is compared with several existing medical image segmentation models, i.e., 3D U-Net [21], V-Net [22], 3D nnU-Net, and Cascade nnU-Net [23]. Since 3D deep learning networks have demonstrated their superiority on volumetric medical image segmentation task [24, 25], the developed network is only compared with these state-of-the-art 3D deep learning-based models.

### 3.1. Internal Test

The quantitative performances based on pixel level of automatic venous thrombus segmentation are summarized in Table 4. All numbers in the table referred to the mean ± standard deviation values of 5-fold cross-validation experiments. Compared with other models, our network provides the best values of DSC (0.76 ± 0.01), AHD (4.11 ± 1.01), and AVD (0.16 ± 0.02).

Table 5 demonstrates the results of quantitative results based on the vessel segment level. Our network outperforms other models in terms of SE (0.95 ± 0.07), SP (0.97 ± 0.02), ACC (0.96 ± 0.03), PPV (0.97 ± 0.04), and NPV (0.97 ± 0.04).

**Figure 3:**
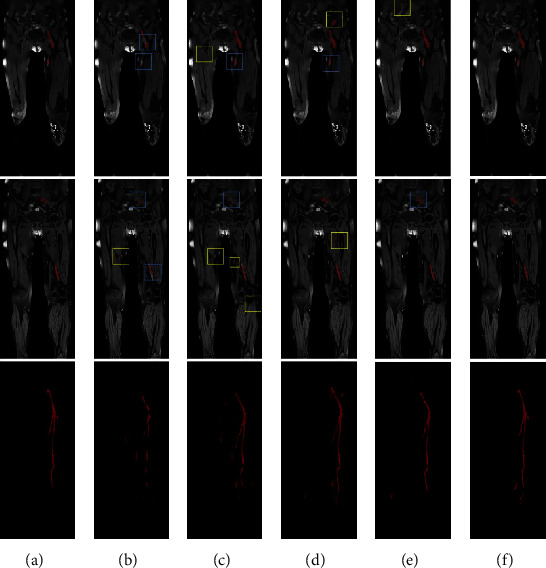
Representative thrombus segmentation results of different networks for a patient. (a) Ground truth, (b) 3D U-Net, (c) V-Net, (d) 3D nnU-Net, (e) Cascade nnU-Net, (f) our proposed network. The first two rows show the segmentation result from the coronal plane, and the last row is to observe the full result directly from the maximum intensity projection (MIP). Red areas in the different models indicate the ground truth and the segmentation result. Yellow boxes highlight some oversegmentation errors. Blue boxes indicate the loss area of DVT segmentation results.

Representative segmentation results are shown in Figure 3. The segmentation results of 3D U-Net can only segment the rough outline of the thrombus and has some segmentation errors on misidentifying other tissue as thrombus and discontinuity segmentation of the thrombus (Figure 3(b)). V-Net performs better than 3D U-Net in eliminating discontinuity (Figure 3(c), blue box) of the result but still misidentifies some tissues as thrombus (Figure 3(c), yellow box). 3D nnU-Net gets much better results than 3D U-Net and V-Net (Figure 3(d)); it achieves excellent scores in DSC, AHD, and AVD. However, 3D nnU-Net also misidentifies iliac and muscle tissues as thrombus (Figure 3(d)). Cascade nnU-Net cascades a low-resolution network and a high-resolution network, but the misidentification problem remains unsolved (Figure 3(e)). Compared to 3D U-Net, V-Net, 3D nnU-Net, and Cascade nnU-Net, our developed network can reduce the segmentation errors and match with the ground truth well (Figure 3(f)).

### 3.2. External Test

**Figure 4:**
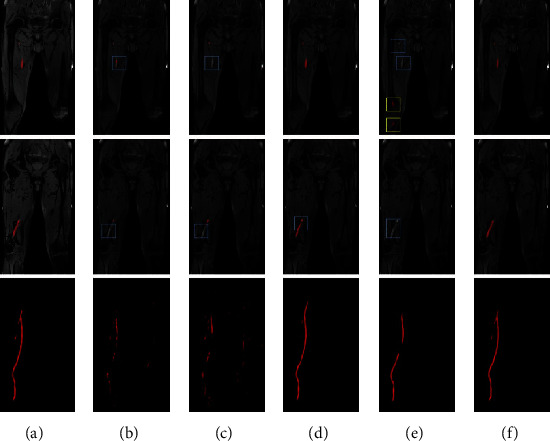
Representative segmentation results of a patient obtained from an external center by DANTE-SPACE techniques. (a) Ground truth, (b) 3D U-Net, (c) V-Net, (d) 3D nnU-Net, (e) Cascade nnU-Net, (f) our proposed network.

For the external test set obtained by DANTE-SPACE on the 3.0 T MR scanner, the segmentation results are shown in Tables 6 and 7. Our proposed network achieves the best results on the venous thrombus segmentation in terms of DSC, AHD, and AVD based on the pixel level, as well as SE, SP, ACC, PPV, and NPV based on the vessel segment level. Representative segmentation results from a patient are shown in Figure 4. Other models may have incomplete segmentation of the thrombus area (Figure 4, blue box) and misidentify the muscle area as thrombus (Figure 4, yellow box); the proposed network has avoided the above errors. The last row of whole lower extremity venous thrombus MIP images shows that the segmentation result of the proposed network can also avoid misidentification errors on 3.0 T DANTE-SPACE images.

**Figure 5:**
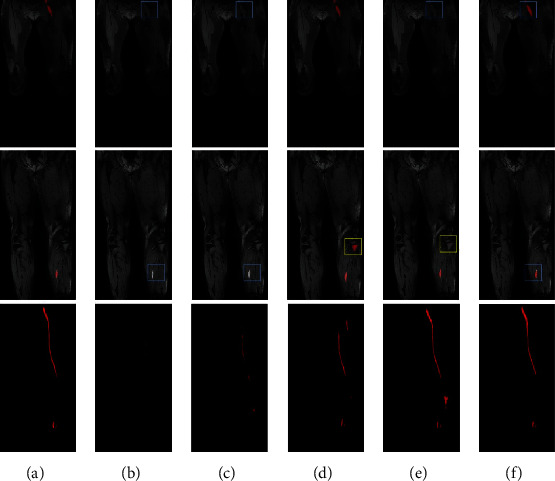
Representative segmentation results of a patient obtained from an external center by DANTE-FLASH techniques. (a) Ground truth, (b) 3D U-Net, (c) V-Net, (d) 3D nnU-Net, (e) Cascade nnU-Net, (f) our proposed network.

For the other external test set obtained by DANT-FLASH on the 3.0 T MR scanner, the segmentation results are shown in Tables 8 and 9. Results suggest that our network remains to achieve the best thrombus segmentation performance compared with other models. Representative segmentation results from a patient are shown in Figure 5. The proposed network has avoided some segmentation problems, such as incomplete segmentation of low-intensity thrombus area (Figure 5, blue box) and segment the popliteal bone area into thrombus (Figure 5, yellow box) in other models. The venous thrombus coronal and MIP segmentation results demonstrate that the proposed network can also match with the ground truth well on the DANTE-FLASH images.

## 4. Discussion

A deep learning network is proposed for automatic venous thrombus segmentation from BTI images with high accuracy and reliability. This network is tested and externally tested by subjects from three different centers and compared with 3D U-Net, V-Net, 3D nnU-Net, and Cascade nnU-Net models. Extensive experimental results based on the pixel level, vessel segment level, and venous thrombus segmentation figures demonstrate that the proposed network achieves superior segmentation performance to state-of-the-art models, indicating the great potential of assisting the DVT diagnosis in clinics.

Deep learning-based medical image segmentation models have achieved unprecedented segmentation performance [26]. However, due to the difficulties described in Figure 1, current deep learning models cannot be directly used for segmenting thrombus from BTI images. These difficulties lead to the most common segmentation error: the tissue with similar thrombus signal intensities and shapes is easily misidentified as thrombus (Figure 3). To address this issue, the proposed network uses 3D segmentation model as the generator and jointly adopts 3D discriminator to supervise generator's performance and enhance the capability of accurate thrombus segmentation, especially to distinguish the thrombus from other similar tissues. Experiment results demonstrated this strategy could reduce the segmentation errors from the abdomen and bone, making the segmentation results closer to the ground truth. Moreover, this proposed network also outperforms other models on the external testing sets in terms of reducing misidentification errors (Figures 4 and 5).

To evaluate the robustness of a neural network, an external test set is necessary [27]. In this work, two kinds of external subjects are tested between different models. One kind of external subject is acquired by DANTE-SPACE on 3.0 T MR scanner, and the other one is acquired by DANTE-FLASH on another 3.0 T MR scanner (Table 1). As the sequences and scanners used for acquiring these external subjects are quite different from those for acquiring training and testing, the image properties (i.e., signal-to-noise ratio and contrast-to-noise ratio) could also be different and may lead to poor segmentation performance on external subjects. Nevertheless, external testing results demonstrate that our proposed network still has the best segmentation performance compared with other models (Table 6–9). The excellent generalization ability of the proposed network due to the following two reasons. Firstly, this network uses the similar segmentation structure of nnU-Net as the generator, and nnU-Net has already proved the generalization ability on dozens of public medical datasets. Moreover, this proposed network uses a discriminator strategy forces model to train a more powerful generator than normal nnU-Net on generalization performance. Secondly, although the image properties of the training and the external testing sets are different, they all belonged to BTI images on which the venous blood is black, and the thrombus is located within the black-blood veins. This is an important prerequisite for excellent generalization performance.

The success of the thrombus segmentation from BTI images can be beneficial to clinical practices. Personalized treatment of DVT patients depends on an accurate diagnosis of the thrombus locations, especially the thrombus signal characteristics. Previous studies demonstrated that the thrombus shape and signal intensity can be used to identify the recurrent DVT, the DVT stage, and the prognosis of DVT treatments [28]. As the thrombus could be automatically segmented from BTI images, quantitative analysis of the thrombus characteristics becomes an easy task.

There are also two limitations in this work. Firstly, the training DVT subjects are still insufficient, and more data should be collected to construct a more robust model. Secondly, because of constraints by long training time, we were not able to perform model embedding experiments to improve the venous segmentation performance [29]. However, as a pilot experiment, the developed network has shown promising results despite all the limitations and will provide insights into future studies.

## 5. Conclusions

A novel deep learning network based on GAN is developed for accurate and automatic thrombus segmentation from BTI images. Experiment results demonstrated that the developed network is more accurate and stable compared with other state-of-the-art models. It has the potential to be an automatic method to assist the diagnosis of DVT in clinics.

## Figures and Tables

**Table 1:** Imaging parameters of DANTE-SPACE performed on 1.5 T MR scanner, DANTE-SPACE performed on 3.0 T MR scanner, and DANTE-FLASH performed on 3.0 T MR scanner.

Parameters	DANTE-SPACE (1.5 T)	DANTE-SPACE (3.0 T)	DANTE-FLASH (3.0 T)
Repetition time (ms)	650	650	5.4
Echo time (ms)	11	9.8	2.08
Turbo factor	40	40	112
Fat suppression	Yes	Yes	Yes
Flip angle	T1 variable	T1 variable	12°
FOV (mm^2^)	352 × 352	352 × 352	380 × 380
Number of partitions	208-256	208-256	208-256
Voxel size (mm^3^)	1.4 × 1.4 × 1.4	1.1 × 1.1 × (1.1 − 1.3)	1.2 × 1.2 × 1.2
Reconstructed voxel size (mm^3^)	0.7 × 0.7 × 0.7	0.5 × 0.5 × (0.5/0.6)	0.6 × 0.6 × 0.6
Bandwidth (Hz/pixel)	698	710	425

**Table 2:** Results of the different loss function based on the proposed network.

Method	DSC	AHD (mm)	AVD
*L*_CE_	0.70 ± 0.09	8.36 ± 5.21	0.26 ± 0.11
*L*_SD_	0.72 ± 0.03	6.52 ± 4.01	0.23 ± 0.10
*L*_CE_ and *L*_SD_	0.73 ± 0.02	5.68 ± 3.24	0.20 ± 0.09
*L*_CE_ and *L*_GAN_	0.73 ± 0.04	6.56 ± 3.23	0.20 ± 0.10
*L*_SD_ and *L*_GAN_	0.72 ± 0.02	5.93 ± 2.38	0.21 ± 0.08
*L*_CE_ and *L*_SD_ and *L*_GAN_	0.75 ± 0.03	5.12 ± 2.13	0.18 ± 0.07

**Table 3:** Representative results of parameter sensitivity analysis of the loss function for the proposed network.

*λ*_1_	*λ*_2_	*λ*_3_	DSC	AHD (mm)	AVD
1.0	1.0	0.5	0.74 ± 0.02	5.52 ± 3.21	0.20 ± 0.08
1.0	1.0	1.0	0.75 ± 0.03	5.12 ± 2.13	0.18 ± 0.07
1.0	1.0	2.0	0.71 ± 0.06	7.42 ± 4.68	0.25 ± 0.12
1.0	0.5	1.0	0.74 ± 0.04	5.43 ± 3.23	0.20 ± 0.10
1.0	0.6	1.0	0.75 ± 0.03	4.86 ± 2.14	0.18 ± 0.06
**0.4**	**0.6**	**1.0**	0.76 ± 0.01	4.11 ± 1.01	0.16 ± 0.02
0.5	0.6	1.0	0.75 ± 0.01	4.63 ± 2.04	0.17 ± 0.05

**Table 4:** The segmentation comparison between different networks based on the pixel level.

Method	DSC	AHD (mm)	AVD
3D U-Net	0.65 ± 0.05	12.07 ± 8.89	0.26 ± 0.09
V-Net	0.63 ± 0.04	16.62 ± 11.05	0.28 ± 0.06
3D nnU-Net	0.76 ± 0.02	5.49 ± 3.10	0.20 ± 0.08
Cascade nnU-Net	0.75 ± 0.03	6.36 ± 3.83	0.22 ± 0.04
Ours	0.76 ± 0.01	4.11 ± 1.01	0.16 ± 0.02

**Table 5:** The segmentation comparison between different networks based on the vessel segment level.

Method	SE	SP	ACC	PPV	NPV
3D U-Net	0.51 ± 0.14	0.76 ± 0.05	0.69 ± 0.05	0.47 ± 0.10	0.78 ± 0.05
V-Net	0.77 ± 0.18	0.83 ± 0.03	0.81 ± 0.07	0.63 ± 0.09	0.90 ± 0.08
3D nnU-Net	0.94 ± 0.07	0.89 ± 0.06	0.91 ± 0.06	0.79 ± 0.12	0.96 ± 0.04
Cascade nnU-Net	0.86 ± 0.14	0.90 ± 0.03	0.89 ± 0.05	0.77 ± 0.09	0.94 ± 0.06
Ours	0.95 ± 0.07	0.97 ± 0.02	0.96 ± 0.03	0.97 ± 0.04	0.97 ± 0.04

**Table 6:** The segmentation comparison between different networks based on the pixel level from 3.0 T DANTE-SPACE.

Method	DSC	AHD (mm)	AVD
3D U-Net	0.57 ± 0.10	25.60 ± 10.69	0.37 ± 0.12
V-Net	0.53 ± 0.13	27.50 ± 11.41	0.45 ± 0.20
3D nnU-Net	0.75 ± 0.09	7.42 ± 4.31	0.20 ± 0.09
Cascade nnU-Net	0.71 ± 0.15	11.20 ± 5.16	0.27 ± 0.11
Ours	0.76 ± 0.06	6.45 ± 3.41	0.18 ± 0.09

**Table 7:** The segmentation comparison between different networks based on the vessel segment level from 3.0 T DANTE-SPACE.

Method	SE	SP	ACC	PPV	NPV
3D U-Net	0.56 ± 0.07	0.54 ± 0.20	0.54 ± 0.19	0.32 ± 0.09	0.82 ± 0.10
V-Net	0.53 ± 0.06	0.40 ± 0.20	0.38 ± 0.20	0.23 ± 0.05	0.77 ± 0.15
3D nnU-Net	0.91 ± 0.08	0.80 ± 0.13	0.85 ± 0.06	0.58 ± 0.10	0.93 ± 0.02
Cascade nnU-Net	0.70 ± 0.28	0.90 ± 0.08	0.84 ± 0.10	0.70 ± 0.24	0.89 ± 0.10
Ours	0.93 ± 0.06	0.92 ± 0.09	0.94 ± 0.05	0.82 ± 0.18	0.96 ± 0.02

**Table 8:** The segmentation comparison between different networks based on the pixel level from 3.0 T DANTE-FLASH.

Method	DSC	AHD (mm)	AVD
3D U-Net	0.42 ± 0.11	57.46 ± 13.58	0.44 ± 0.17
V-Net	0.38 ± 0.14	75.33 ± 12.91	0.65 ± 0.21
3D nnU-Net	0.73 ± 0.13	7.27 ± 5.42	0.24 ± 0.13
Cascade nnU-Net	0.68 ± 0.16	13.56 ± 7.63	0.38 ± 0.15
Ours	0.73 ± 0.11	6.49 ± 3.57	0.22 ± 0.12

**Table 9:** The segmentation comparison between different networks based on the vessel segment level from 3.0 T DANTE-FLASH.

Method	SE	SP	ACC	PPV	NPV
3D U-Net	0.53 ± 0.29	0.68 ± 0.18	0.65 ± 0.15	0.41 ± 0.15	0.81 ± 0.12
V-Net	0.57 ± 0.25	0.59 ± 0.17	0.57 ± 0.15	0.33 ± 0.17	0.78 ± 0.14
3D nnU-Net	0.81 ± 0.14	0.88 ± 0.09	0.89 ± 0.05	0.72 ± 0.21	0.93 ± 0.06
Cascade nnU-Net	0.64 ± 0.17	0.83 ± 0.04	0.79 ± 0.04	0.52 ± 0.17	0.88 ± 0.06
Ours	0.81 ± 0.12	0.97 ± 0.02	0.95 ± 0.04	0.96 ± 0.10	0.94 ± 0.05

## Data Availability

The black blood MR data used to support the findings of this study are currently under embargo while the research findings are commercialized. Requests for data, 6 months after publication of this article, will be considered by the corresponding author.
